# Neurofilament light chain as biomarker for axonal damage in Guillain-Barré syndrome

**DOI:** 10.1136/jnnp-2020-324308

**Published:** 2020-11-05

**Authors:** Bart C Jacobs

**Affiliations:** Neurology and Immunology, Erasmus Medical Center, Rotterdam, The Netherlands

**Keywords:** guillain-barre syndrome

## A new tool to monitor disease activity and predict outcome

Axonal degeneration is an important determinant of poor outcome in the Guillain-Barré syndrome (GBS). Axonal injury may occur in acute motor axonal neuropathy (AMAN) as well as acute inflammatory demyelinating polyneuropathy and in severe cases may result in irreversible degeneration. Treatment with immunoglobulins or plasma-exchange aims to prevent further axonal injury, but clinicians currently have no methods to monitor the response directly. Clinical deficits only partly reflect the axonal damage and depend on other causes of nerve dysfunction, including demyelination. Nerve conduction studies may be normal or changeable in the early phase and require considerable expertise. We have prognostic models based on clinical features, but their performance could be improved by biomarkers for early axonal injury. One of the most promising prognostic biomarkers in a variety of neurological diseases is neurofilament light chain (NfL), a protein which is exclusively present in neuronal cytoplasm.^1^ NfL has been reported as biomarker already two decades ago, but recent single-molecule array technology enables the accurate detection of NfL at ultralow levels in blood for longitudinal studies.

In the study published in *Journal of Neurology, Neurosurgery and Psychiatry*, Martín-Aguilar and coworkers provide compelling evidence that serum NfL levels predict the long-term outcome in GBS.^2^ They have examined serum from 98 patients with GBS included by the Spanish hospitals participating in the ongoing prospective International GBS Outcome Study.^3^ In patients with GBS, the median NfL level in serum obtained at hospital admission was five times higher than in age-matched healthy controls, and returned to normal after 1-year follow-up. Serum NfL varied considerably between patients, ranging from normal to 100-fold increased levels, and high levels were related to higher disability and AMAN. Moreover, serum NfL levels predicted the ability to run independent of age, GBS disability score and Medical Research Council (MRC)-sumscore, indicating that NfL may be used to improve current prognostic models.

As for all good studies, the results also raises new questions. Can NfL be used to monitor progression of axonal injury in individual patients to identify the opportunities for additional treatment? Such monitoring could be especially useful in patients with tetraparalysis, treatment-related fluctuations and transition to chronic inflammatory demyelinating polyneuropathy. NfL may also be a potential secondary outcome measure to evaluate new treatments for GBS. Can NfL add to the ongoing debate on the criteria for axonal and demyelinating subtypes of GBS, although such studies may also indicate that these subtypes are not as distinct as previously thought? NfL in cerebrospinal fluid may be a potential biomarker for the involvement of nerve roots in GBS. More extensive studies are required to decide if NfL truly adds to the existing clinical prognostic models in GBS. The authors already defined cut-off values for NfL as a single predictor, but more likely in my view is that NfL will be combined with clinical features to reach most predictive power. NfL is probably the first of many biomarkers for peripheral nerve injury that can be measured by the current ultrasensitive technologies. This study is an important step towards a new era where biomarkers are used to improve the management of GBS and other inflammatory neuropathies.
